# Supplementary material for: Depletion of Lipocalin 2 (LCN2) in Mice Leads to Dysbiosis and Persistent Colonization with Segmented Filamentous Bacteria
Source: Int J Mol Sci. 2021 Dec 5;22(23):13156. doi: 10.3390/ijms222313156 (PMC8658549; doi:10.3390/ijms222313156)

International Journal of *Molecular Sciences*

MDPI

Supplements

Depletion of Lipocalin 2 (LCN2) in Mice Leads to Dysbiosis and Persistent Colonization with Segmented Filamentous Bacteria

Patrick Klüber [1,†], Steffen K. Meurer [2,†], Jessica Lambertz [2,†], Roman Schwarz [3], Silke Zechel-Gran [1], Till Braunschweig [4], Sabine Hurka [5], Eugen Domann [6,*] and Ralf Weiskirchen [2,*]

Table S1. Mouse primers used in real-time quantitative PCR.

Gene	Acc. No./Ref.	Primer
Lcn2	NM_008491.1	For: 5′-ccatctatgagctacaagagaacaat-3′<br>Rev: 5′-tctgatccagtagcgacagc-3′
T-bet	NM_019507.2	For: 5′ -aagttcaaccagcaccagaca-3′<br>Rev: 5′ -cacggtgaaggacaggaatgg-3′
Tgf-β1	NM_011577.2	For: 5′ -tggagcaacatgtggaactc-3′<br>Rev: 5′ -cagcagccggttaccaag-3′
Gata3	NM_008091.3	For: 5′-cccattaccacctatccgcc -3′<br>Rev: 5′-gttcacacactccctgcctt -3′
Il-5	NM_010558.1	For: 5′ -atgaggcttcctgtccctact-3′<br>Rev: 5′ -tacccccacggacagtttga-3′
Il-13	NM_008355.3	For: 5′ -ggtatggagtgtggacctgg-3′<br>Rev: 5′ -gccatgcaatatcctctgggt-3′
Tnf-a	NM_013693.3	For: 5′ -accacgctcttctgtctactga-3′<br>Rev: 5′ -tccacttggtggtttgctacg-3′
Ifn-γ	NM_008337.4	For: 5′ -ggaggaactggcaaaaggatgg-3′<br>Rev: 5′ -tgttgctgatggcctgattgtc-3′
Il-1β	NM_008361.4	For: 5′ -gagctgaaagctctccacctc-3′<br>Rev: 5′ -ctttcctttgaggcccaaggc-3′
Mcpt1	NM_008570.1	For: 5′ -gcacttctcttgccttctgg-3′<br>Rev: 5′ -taaggacgggagtgtggtct-3′
Mcpt2	NM_008571.1	For: 5′ -gcacttcttttgccttctgg-3′<br>Rev: 5′ -taaggacgggagtgtggttt-3′
Mcpt6 (Tpsb2)	NM_010781.3	For: 5′ -tgctgtgtgctggaaatacc-3′<br>Rev: 5′ -cccttcactttgcagacca-3′
Nos2	NM_010927.4	For: 5′-tgccccttcaatggttggt-3′<br>Rev: 5′-tccttcggcccacttcct-3′
RegIIIγ	NM_011260.2	For: 5′-ccttcctcttcctcaggcaat-3′<br>Rev: 5′-taattctctctccacttcagaaatcct-3′
Saa1	NM_009117.4	For: 5′-catttgttcacgaggctttcc-3′<br>Rev: 5′-gtttttccagttagcttccttcatgt-3′
Saa2	NM_011314.3	For: 5′-tgtgtatcccacaagggtttcaga-3′<br>Rev: 5′-ttattaccctctcctcctcaagca-3′
Il-17a	NM_010552.3	For: 5′-ggactctccaccgcaatga-3′<br>Rev: 5′-ggcactgagcttcccagatc-3′
Il-21	NM_001291041.1	For: 5′-catcattgacctcgtggccc-3′<br>Rev: 5′-acgaatcacaggaagggcat-3′
Il-22	NM_016971.2	For: 5′-ggtgacgaccagaacatcca-3′<br>Rev: 5′-cagttccccaatcgccttga-3′
Rorc	NM_001293734,1	For: 5′-ggaggacagggagccaagtt-3′<br>Rev: 5′-ccgtagtggatcccagatgact-3′
Foxp3	NM_001199347.1	For: 5′-agaggtattgagggtgggtgt-3′<br>Rev: 5′-cagcatgggtctgtcttctcta-3′
Gapdh	NM_008084.3	For: 5′-tgttgaagtcacaggagacaacct-3′<br>Rev: 5′-aacctgccaagtatgatgacatca-3′
β-actin	NM_007393.5	For: 5′ -ctctagacttcgagcaggagatgg-3′<br>Rev: 5′ -atgccacaggattccatacccaaga-3′

Table S2. Overview of the four experimental animal groups used for microbiome analysis.

Group	Genotype[1]	Gender	Number (n)	Age (weeks)
1	WT	female	5	6
2	WT	male	3	12
3	*Lcn2*$^{-/-}$	female	3	4, 6, 9
4	*Lcn2*$^{-/-}$	male	2	7

[1] Abbreviations used are: *Lcn2*$^{-/-}$, deficient for Lipocalin 2; WT, wild type

Table S3. Composition of the diet.

Ingredient	Composition
Crude Nutrients [%]	Dry matter (87.7); Crude protein (19.0); Crude fat (3.3); Crude fibre (4.9); Crude ash (6.4); N free extracts (54.1); Starch (36.5); Sugar (4.7)
Minerals [%]	Calcium (1.0); Phosphorus (0.70); Sodium (0.24); Magnesium (0.22); Potassium (0.91)
Fatty acids [%]	C 14:0 (0.01); C 16:0 (0.47); C 16:1 (0.01); C 18:0 (0.08); C 18:1 (0.62); C 18:2 (1.80); C 20:0 (0.01); C 20:1 (0.02)
Amino acids [%]	Lysine (1.00); Methionine (0.30); Methionine +Cysteine (0.65); Threonine (0.68); Tryptophan (0.25); Arginine (1.14); Histidine (0.44); Valine (0.88); Isoleucine (0.76); Leucine (1.30); Phenylalanine (0.85); Phenylalanine +Tyrosine (1.43); Glycine (0.80); Glutamic acid (3.90); Aspartic acid (1.61); Proline (1.25); Alanine (0.79); Serine (0.89)
Vitamins (per kg)	Vitamin A (15,000 IU); Vitamin D3 (1,000 IU); Vitamin E (110 mg); Vitamin E as menadione (5 mg); Thiamine (18 mg); Riboflavin (23 mg); Pyridoxine (21 mg); Cobalamin (100 µg); Nicotinic acid (135 mg); Pantothenic acid (43 mg); Folic acid (7 mg); Biotin (525 µg); Choline-chloride (2,990 mg); Inositol (100 mg)
Trace elements (per kg)	Iron (179 mg); Manganese (69 mg); Zinc (94 mg); Copper (16 mg); Iodine (2.2 mg); Selenium (0.3 mg); Cobalt (2.1 mg)

Table S4. Listing and combination of the index primers used. Sample assignment is based on animal groups 1-4 with the corresponding sample identification.

Sample	Sample assignment	Index primer 1	Index primer 2
Wild type	1.1	N 716	S 511
	1.2	N 718	S 511
	1.3	N 719	S 511
	2.1	N 720	S 511
	2.2	N 721	S 511
	2.3	N 722	S 511
	2.4	N 723	S 511
	2.5	N 724	S 511
	2.6	N 726	S 511
	2.7	N 727	S 511
Lcn2$^{-/-}$	3.1	N 728	S 511
	3.2	N 729	S 511
	3.3	N 716	S 510
	3.4	N 718	S 510
	3.5	N 719	S 510
	3.6	N 720	S 510
	3.7	N 721	S 510
	4.1	N 722	S 510
	4.2	N 723	S 510
	4.3	N 724	S 510
	4.4	N 726	S 507
	4.5	N 727	S 507
Controls	(-) reaction mix (DNA extraction)	N 728	S 507
	(-) no template control 16S rRNA PCR	N 729	S 507
	(+) chromosomal DNA *E. coli* 16S rRNA PCR	N 716	S 507
	(+) mock community 16S rRNA PCR	N 718	S 507

Table S5. Bacterial primers used in PCR.

Gene	Designation	Primer[1]
V3-region 16S rRNA	341-F	For: 5′-attaccgcggctgctgg-3′
V3-region 16S rRNA	534-R	Rev: 5′-cctacgggaggcagcag-3′
SFB 16S rRNA	779-F	For: 5′-tgtgggttgtgaataacaat-3′
SFB 16S rRNA	1008-R	Rev: 5′-gcgggcttccctcattacaagg-3′
SFB 16S rRNA	1380-R	Rev: 5′-ggttagcccacaggcttcgg-3′

[1] All primer sequences were obtained from Yin *et al.*, *ISME J.* **2013**, *7*, 615-21.

Figure S1. Relative share of genera [% of total reads] at sample level

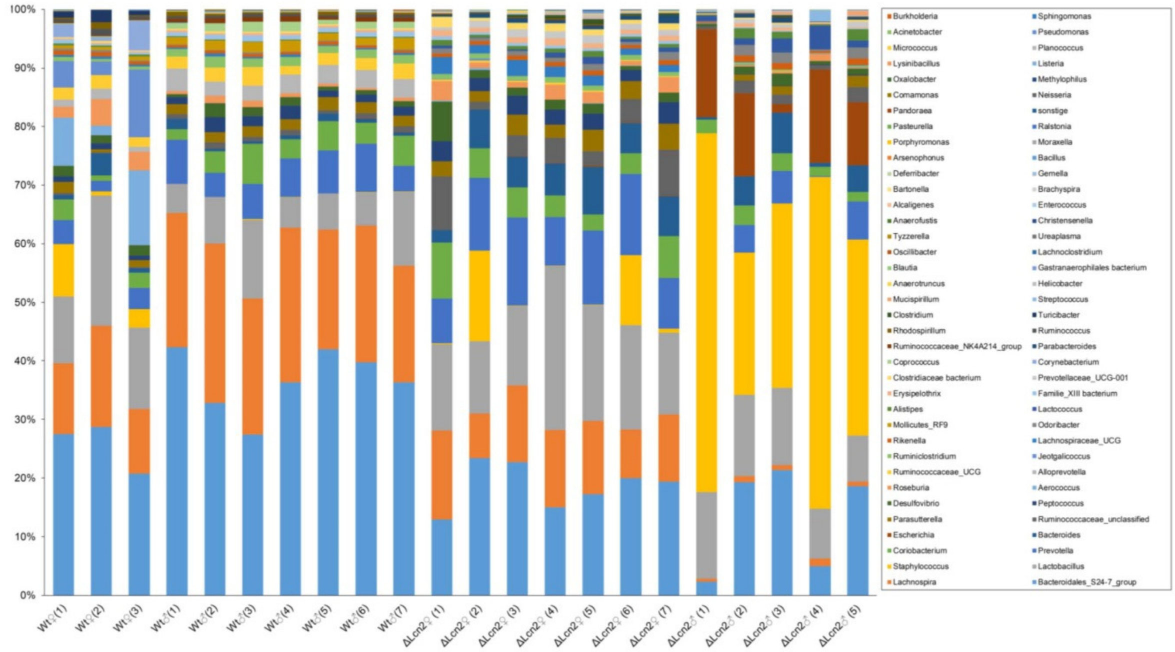

Figure S2. Shannon index rarefaction curves at the sample level

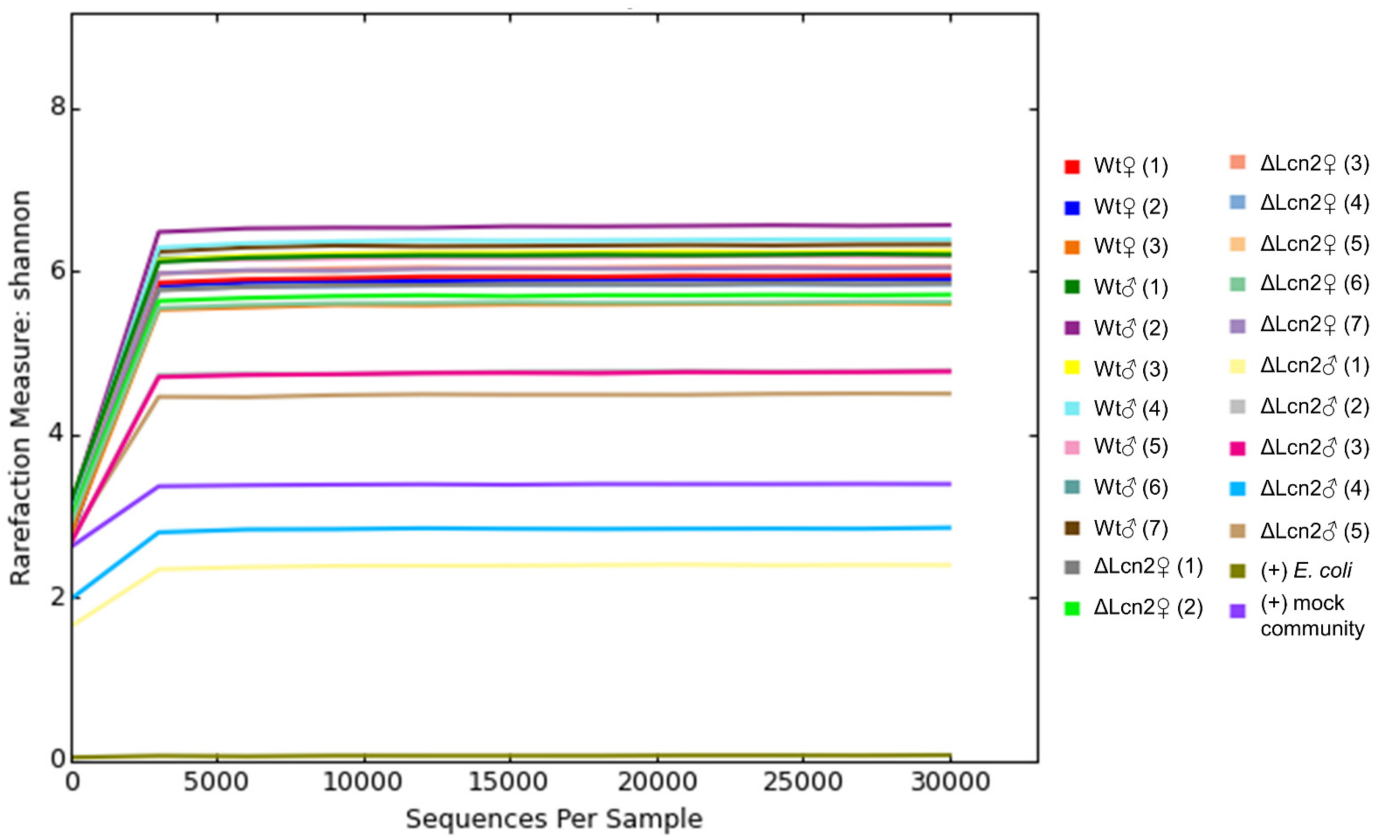

Supplement: Supplementary file 1 [file ijms-22-13156-s001.zip › ijms-1462645-supplementary.pdf]
